# A Norm-Based Conditional Process Model of the Negative Impact of Optimistic Bias on Self-Protection Behaviors During the COVID-19 Pandemic in Three Chinese Cities

**DOI:** 10.3389/fpsyg.2021.659218

**Published:** 2021-06-07

**Authors:** Sijing Chen, Jianwei Liu, Huamin Hu

**Affiliations:** School of Economics and Management, Zhejiang University of Science and Technology, Hangzhou, China

**Keywords:** optimistic bias, social norms, self-protection behavior, message acceptance, COVID-19

## Abstract

Data were collected from 896 participants in three Chinese cities affected by the COVID-19 pandemic to varying degrees through an online survey platform. A conditional process model was then proposed for the impact of optimistic bias on self-protection behaviors during the COVID-19 pandemic from the perspective of social norms. Statistical analysis demonstrates that optimistic bias has a negative impact on self-protection behaviors through message acceptance. Perceived social norms moderate this relationship in the following ways: (1) The higher the perceptions of social norms, the smaller the negative impact of optimistic bias on message acceptance, and the smaller the positive impact of message acceptance on self-protection behaviors. (2) Within a certain range, the higher the perceptions of social norms, the smaller the negative impact, both direct and indirect, of optimistic bias on self-protection behaviors. (3) The direct and indirect effects of optimistic bias on self-protection behaviors become insignificant when perceptions of social norms are very strong. Comparing the data of the three cities shows that higher risk is associated with a stronger role of social norms in moderating the relationship between optimistic bias and self-protection behaviors. The above results suggest that there may be both internal (optimistic bias) and external (social norms) reference points in individual decision-making regarding health behaviors. The theoretical and practical significance of the dual reference points are discussed.

## Introduction

Cable News Network (CNN) reported on March 25, 2020 that a group of young adults who thought “they were invincible” held a coronavirus party in Kentucky, the United States, to defy state guidance to practice social distancing, and that at least one of them were then found to have the novel coronavirus (SARS-CoV-2)^1^ In fact, the World Health Organization (WHO) has warned young people that they are not invincible from the novel coronavirus^2^ The overconfidence of the partygoers in their immunity is exactly what psychologists call optimistic bias or unrealistic optimism; that is,people systematically tend to underestimate (overestimate) their personal probability of encountering negative (positive) events compared with other individuals under the same conditions (Weinstein, 1980; Harris and Hahn, 2011). In subsequent studies, the concept of optimistic bias has been widely used in various domains, one of which is health-related behavior (e.g., Williams and Clarke, 1997; Arnett, 2000; Caponecchia, 2010; Lopez and Leffingwell, 2020). The existing studies in this field can be broadly categorized into three groups. First, studies that examine the impact of optimistic bias on health behavior in different behavioral areas, such as alcohol consumption (Masiero et al., 2018), smoking (Popova and Halpern-Felsher, 2016), sun protection (Bränström et al., 2006), obesity and hypertension (White et al., 2017), and safe driving (Delhomme et al., 2009). Most of these studies concluded that optimistic bias had a negative impact on health behavior (Weinstein and Klein, 1995; Harris and Napper, 2005; Park and Ju, 2016; Hwang et al., 2019). However, some authors have expressed different views; for example, Cho et al. (2013) found that optimistic bias had no significant effect on self-protection behavior during H1N1 influenza pandemic in South Korea; Taylor and Gollwitzer (1995) also suggested a non-significant association between optimistic bias and behavior. Second, studies that investigate the moderators in the relationship between optimistic bias and health behavior (Helweg-Larsen and Shepperd, 2001). For example, Harris et al. (2008) indicated that event characteristics (e.g., the universality, negativity, or severity of the event) and personal factors (e.g., emotional state and past experience) moderated the impact of optimistic bias to varying degrees. Third, studies that aim at intervening optimistic bias. The purpose of this group of studies is to explore how to reduce the optimistic bias of participants, thereby mitigating the negative impact on health behavior. The intervention methods that have received considerable attention include self-affirmation (Klein et al., 2010; Epton et al., 2015), perceived control (Jansen et al., 2018), and self-efficacy (Morisset et al., 2010).

Despite their different focuses, all the above three groups of studies examine the relationship between optimistic bias and health behavior from an individual perspective, without considering the social influence, defined as change in a person's cognition, attitude, or behavior that results from observation of or interaction with others (Raven, 1964), as Nolan et al. (2008) pointed out, social influence is often underestimated. Individuals' behaviors and attitudes, including health behavior, are influenced to a large extent by those of others in social situations (Cialdini et al., 1991). In fact, individual perception of health risks, including judgment about the chance of developing a disease for others and themselves, is affected by the information about how most people behave in a given situation (Liao et al., 2011; Dempsey et al., 2018; Limbu et al., 2018), i.e., information based on social norms (Jiang et al., 2009). In fact, the influence of social norms on health behaviors has attracted much attention (Thomas et al., 2016; Dempsey et al., 2018; Hang et al., 2020), and has been investigated using different theoretical models, such as the theories of planned behavior (Ajzen, 1991) and normative social behavior (Rimal and Real, 2005). However, no research has been conducted to examine the relationship between optimistic bias and health behavior from the perspective of social norms, which may hinder a better understanding of how optimistic bias affects health behavior (Cho et al., 2013).

Social norms are generally defined as codes of conduct that are different from the laws and regulations and are generally accepted by group members (Cialdini and Trost, 1998). Social norms can be divided into descriptive and injunctive norms. Descriptive norms refer to the perceived prevalence of a behavior, whereas injunctive norms refer to the perceived degree of approval for the behavior (Cialdini et al., 1991). According to Dempsey et al. (2018), the social norm approach (SNA) was first described by Perkins and Berkowitz (1986) in a study of alcohol use among college students. They found that college students generally overestimated alcohol use by peers, which resulted in misperceived descriptive and injunctive norms regarding drinking on campus; that is, they overestimated the drinking of others and the degree of approval for drinking among others. A reasonable coping strategy is to provide individuals with real normative information, thereby reducing normative misperceptions and improving the corresponding behaviors (Blanton et al., 2008). Subsequent studies also found systematic overestimation of negative behaviors of others in other areas, such as smoking (Pischke et al., 2015), distracted driving (Carter et al., 2014), and unsafe sex (McAlaney and Jenkins, 2017). On the contrary, there is evidence that people often underestimate the frequency of positive behaviors or the degree of approval for positive behaviors among others. For example, Lally et al. (2011) found that British teenagers generally overestimated the intake of snacks or sugar-sweetened drinks by peers, but underestimated their daily intake of fruits and vegetables. Reid and Aiken (2013) also reported that participants systematically underestimated the degree to which others took sun protection measures. Providing normative information about the true behavior of others to people who underestimate the positive behavior or overestimate the negative behavior of others can often correct their misperceptions and the corresponding behaviors to varying degrees (e.g., Croker et al., 2009; Reid and Aiken, 2013). Therefore, SNA has become one of the most widely used behavioral intervention techniques (McAlaney et al., 2011; Dempsey et al., 2018). SNA has been studied in many fields. For example, Allcott (2011) analyzed the role of social norms in residential energy conservation, and pointed out that intervention with social norm information significantly reduced residential electricity consumption. Ferraro and Price (2013) found that the provision of social norms information led to a significant reduction in residential water consumption, which was equivalent to that caused by a price increase of 12 to 15% and remained even after 2 years. Ng et al. (2020) argued that normative information affected people's attitude and consequently their willingness to vaccinate against seasonal influenza.

**Figure 1:**
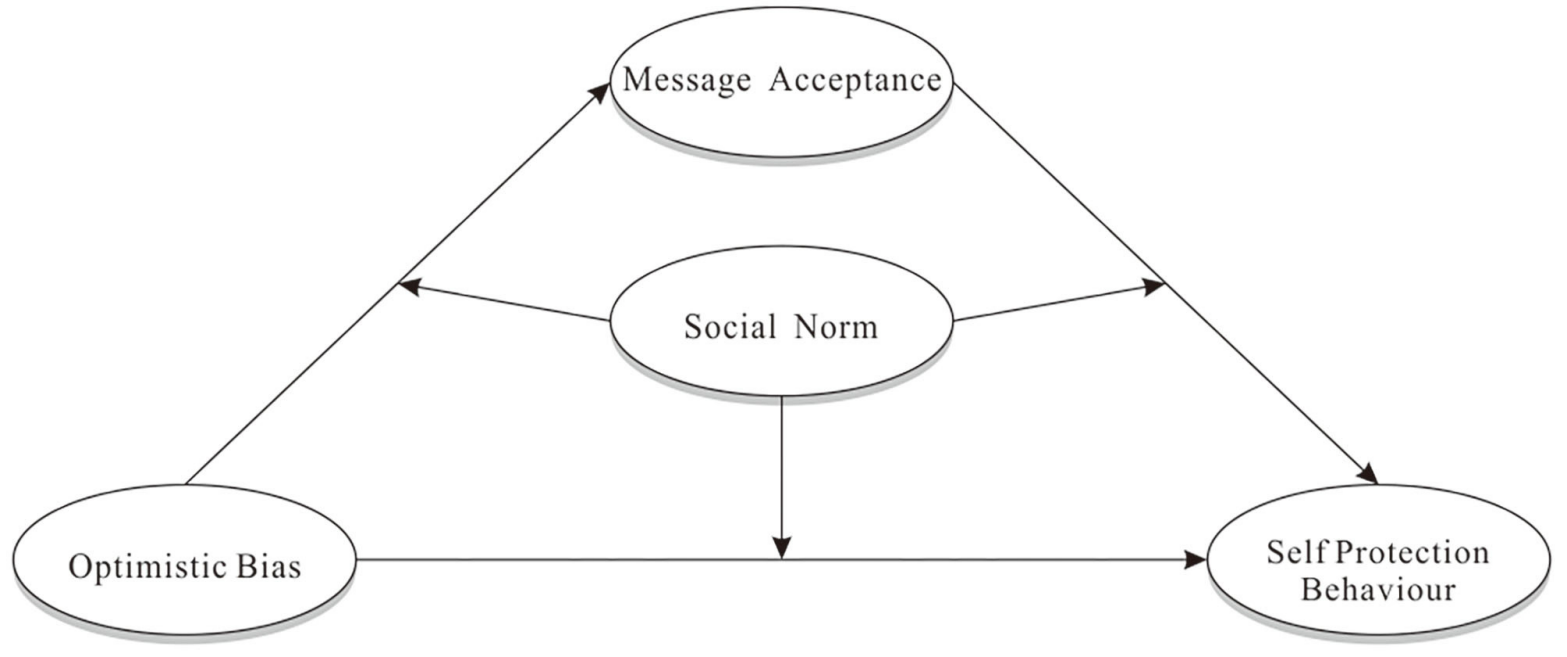
The conditional process model of optimistic bias affecting self-protection behaviors.

SNA provides a new perspective for understanding the impact of optimistic bias on health behavior. Similar to normative misperceptions, optimistic bias is manifested in overestimating (underestimating) the probability of others (themselves) encountering negative events. Some researchers believe that optimistic bias is a stable trait (Weinstein and Klein, 1995; Radcliffe and Klein, 2002; Cho et al., 2013). For example, Helweg-Larsen (1999) noted that people sometimes adjusted their optimistic beliefs due to personal experiences (e.g., earthquakes); however, these changes only lasted for a short period of time as their optimistic bias would quickly return to the previous level. Some researchers suggest that this may be because the optimistic bias may be related to certain personal traits (e.g., trait anxiety) or coping style (Butler and Mathews, 1987; Myers and Brewin, 1996). Therefore, optimistic bias can be regarded as an internal reference point in health behavior decisions. Lü and Zhao (2017) suggested that message acceptance mediated the relationship between optimistic bias and health behavior; that is, optimistic bias negatively affects health behavior by reducing the acceptance of health information. Similarly, Harris et al. (2008) argued that optimistic bias was an obstacle to the acceptance of health information. Perceived social norms often play a role in the impact of optimistic bias on health behavior through message acceptance. Nabi (2015) found that perceptions of social norms affected message acceptance, and that individuals tended to accept message consistent with social norms. It could therefore be speculated that that perceived social norms moderate the relationship between optimistic bias and message acceptance. In addition, Voisin et al. (2016) indicated that when the information contained in the intervention was consistent with certain social norms, it could effectively promote health behavior. Kiviniemi et al. (2018) also noticed that presenting tumor marker information to participants had a significant positive impact on their health behavior, but only when they believed that there were certain social norms. Based on the findings of previous studies, a conditional process model of optimistic bias, message acceptance, and health behavior is proposed from the perspective of social norms. As shown in Figure 1, optimistic bias affects health behavior through message acceptance, while social norms moderate the relationships between optimistic bias and message acceptance and between message acceptance and health behavior. In addition, in order to better understand the relationship between the above variables, this study also examined whether optimistic bias directly affects health behavior and whether perceived social norms also play a moderating role in this path. The proposed model was tested using the self-protection behaviors of individuals during the COVID-19 pandemic as the outcome variable. Considering regional differences in the COVID-19 pandemic, data were collected from three cities with different risk levels to test the proposed model.

To sum up, the main purpose of this study is to explore how perceived social norms affect the relationship between optimistic bias and health behavior, in order to provide a new theoretical perspective for a more comprehensive understanding of this relationship and fill the theoretical gap. Moreover, the similarities and differences of the model between the three cities will be analyzed to better understand how social norms moderate the impact of optimistic bias on self-protection behaviors. Accordingly, the following hypotheses are proposed:

**H1**: There is a negative correlation between optimistic bias and self-protection behavior.
**H2**: Message acceptance mediates the relationship between optimistic bias and self-protection behavior.
**H3**: Perceptions of social norms moderate the relationship between optimistic bias and self-protection behavior.
**H4**: Perceptions of social norms have different moderating effects in areas with different risk levels.

## Methods

### Sample

On February 7, 2020, 1,000 questionnaires were distributed to three Chinese cities, Wuhan, Hangzhou, and Jinan through an online survey platform, So Jump. A total of 896 valid questionnaires were collected, with a response rate of 89.6%. According to the National Health Commission of the People's Republic of China, there were 11,618, 156, and 39 confirmed cases of COVID-19 in Wuhan, Hangzhou, and Jinan, respectively, as of the date of questionnaire distribution (10:00 am on February 7, 2020), roughly corresponding to the high, medium, and low risk levels. The demographics of participants are shown in Table 1. Participants from Wuhan accounted for 31.92% (age *M* = 29.31, *SD* = 11.08), Hangzhou 33.71% (age *M* = 30.34, *SD* = 13.16), and Jinan 34.37% (age *M* = 31.51, *SD* = 13.91). Females accounted for 51.12% of the total sample. Participants with a bachelor's degree or above accounted for 64.40%. This study was approved by the ethics committee of School of Economics and Management, Zhejiang University of Science and Technology and informed consent was obtained from all participants before the survey started.

**Table 1:** Demographics of participants.

**Variables**	**Wuhan**		**Hangzhou**		**Jinan**		**Pooled sample**	
	***N***	**%**	***N***	**%**	***N***	**%**	***N***	**%**
**Sex**								
Male	128	44.76	153	50.66	157	51.31	438	48.88
Female	158	55.24	149	49.34	151	48.69	458	51.12
**Education**								
Less than a bachelor's degree	90	31.47	73	24.17	93	30.19	256	28.57
Bachelor's degree and above	196	68.53	229	75.83	215	69.81	640	71.43
**Occupation**								
Healthcare workers	20	6.99	22	7.28	19	6.17	61	6.81
Non-healthcare workers	266	93.01	280	92.72	289	93.83	835	93.19
Total	286	31.92	302	33.71	308	34.37	896	100

### Measures

The measure of optimistic bias was adapted from Arnett (2000). Participants were asked to rate how likely others (Cronbach's α = 0.86) and themselves (Cronbach's α = 0.86) were to (1) be infected with COVID-19 and (2) get sick from COVID-19 on a 7-point scale, where 1 = extremely unlikely and 7 = extremely likely. The responses to the two items are averaged into one score and optimistic bias is operationalized as the difference between the score for themselves and others. The measure of message acceptance (Cronbach's α = 0.90) was adapted from Harris and Napper (2005). Participants were asked on a 7-point scale from 1 (completely disagree) to 7 (completely agree): “To what extent do you believe that (1) measures recommended by the government can effectively reduce the risk of infection; (2) these recommendations have scientific basis; (3) failure to follow the recommendations increases the risk of infection; and (4) these recommendations are based on true and reliable information.” The measure of self-protection behaviors (Cronbach's α = 0.80) was adapted from Lü et al. (2010) and participants rated five items on a 7-point scale (1 = completely disagree, 7 = completely agree): “(1) I have reduced time spent outside the home; (2) I wear a mask outside; (3) I canceled family gatherings and other gatherings; (4) I follow other government recommendations to reduce the risk of infection; and (5) I recommend people around me to follow government recommendations.” The measure of perceptions of social norms (Cronbach's α = 0.87) was adapted from Liao et al. (2019) and included three items: “My family/friends/most people around me follow government recommendations.” All the above ratings are based on a 7-point scale, where 1 = completely disagree and 7 = completely agree. “Knowledge” (Brug et al., 2004), which is theoretically unrelated to the above variables, was used as a marker variable (Cronbach's α = 0.90), which included three 7-point items (1 = completely disagree, 7 = completely agree): “(1) I am very aware of the mortality of COVID-19; (2) I think I know enough about COVID-19; and (3) I have sufficient knowledge related to COVID-19.” To ensure the sensitivity of testing common method bias (CMB), the marker variable was presented and scored in the same way as other variables, and all items, including those of the marker variable, were arranged in random order.

### Statistical Analysis

First, the mediating effect of message acceptance between optimistic bias and self-protection behavior was examined by traditional three-step regression analysis using SPSS 25.0. Next, the moderating effect of perceptions of social norms in this mediating effect was investigated by the bootstrap method using PROCESS 3.5 developed by Preacher and Hayes (2004) to analyze the direct and indirect effects of optimistic bias on self-protection behavior under different levels of social norm perceptions. Finally, the differences in this moderating effect in cities with different risk levels were analyzed by a secondary moderating model using PROCESS 3.5.

## Results

### Pilot Study

A pre-survey was conducted among 120 college students before the actual survey. The results show that the scales of the five main variables (optimistic bias: perceived own risk, Cronbach's α = 0.85; perceived others' risk, Cronbach's α = 0.90; message acceptance, Cronbach's α = 0.94; self-protection behavior, Cronbach's α = 0.85; perceptions of social norms, Cronbach's α = 0.89; and knowledge, Cronbach's α = 0.88) measured in the questionnaire have high reliability. The measurement model containing the above 6 scales showed an acceptable fit, as demonstrated by confirmatory factor analysis (CFA) using Mplus 8.3 (χ^2^/*df* = 1.68, CFI = 0.942, TLI = 0.930, RMSEA = 0.075, SRMR = 0.056).

### Common Method Bias

Data were collected from the three cities using self-reported questionnaires. The common method bias was minimized by anonymous collection and random arrangement of questions, and assessed by SPSS 25.0 using Harman one-factor analysis. The results showed that 32.91% of the variance was attributed to the first (largest) factor, which was lower than the threshold of 40%, indicating no significant common method bias. Given that the Harman one-factor analysis is insensitive to changes in common method variance (CMV) and CMB (Williams et al., 2010; Tehseen et al., 2017), the CFA marker technique was employed in Mplus 8.3. The results showed no significant differences between the baseline model and models C (Δχ^2^/*df* = 0.40, *p* = 0.527) and U (Δχ^2^/*df* = 0.90, *p* = 0.585). Therefore, it can be ascertained that there was minimal or no common method bias.

### Conditional Process Model

Table 2 describes the mean, standard deviation, and correlation coefficient of each variable. It can be seen that self-protection behavior is significantly positively correlated with message acceptance and social norms, and significantly negatively correlated with optimistic bias. Thus, H1 is confirmed.

**Table 2:** Descriptive statistics and correlation coefficients of variables.

**Variables**	***M***	***SD***	**OB**	**MA**	**SN**	**SB**
Optimistic bias (OB)	1.04	1.58				
Message acceptance (MA)	6.45	0.95	−0.18^**^			
Social norms (SN)	6.30	1.01	−0.15^**^	0.68^**^		
Self-protection behavior (SB)	6.53	0.80	−0.24^**^	0.79^**^	0.71^**^	
Marker variable	4.94	1.81	−0.002	0.06	0.06	0.02

*N = 896,*

** *p < 0.01*.

The mediating effect of message acceptance and the moderating effect of perceived social norms were assessed with self-protection behavior as the dependent variable and optimistic bias as the independent variable using PROCESS 3.5^3^. All variables were centralized to reduce multicollinearity. The results are shown in Table 3. First, the mediating role of message acceptance was examined. The regression coefficients of optimistic bias on both message acceptance and self-protection behavior are significant (*B* = −0.11, β = −0.18, *SE* = 0.02, *p* < 0.001, *R*^2^ = 0.03; *B* = −0.12, β = −0.24, *SE* = 0.02, *p* < 0.001, *R*^2^ = 0.06). In multiple regression, the regression coefficients of optimistic bias (*B* = −0.05, β = −0.10, *SE* = 0.01, *p* < 0.001) and message acceptance (*B* = 0.12, β = 0.77, *SE* = 0.02, *p* < 0.001) on self-protection behavior are significant (*R*^2^ = 0.63). It indicates that message acceptance partially mediates the impact of optimistic bias on self-protection behavior. Therefore, hypothesis 2 is supported.

**Table 3:** Test of conditional process model.

**Variables**	**M**_****1****_ **(dependent variable: message acceptance)**					**M**_****2****_ **(dependent variable: self-protection behavior)**				
	**Coefficients**	***SE***	***t***	**LLCI**	**ULCI**	**Coefficients**	***SE***	***t***	**LLCI**	**ULCI**
Constant	0.01	0.02	0.54	−0.03	0.06	0.05^**^	0.01	3.11	0.02	0.07
OB	−0.04^*^	0.01	−2.40	−0.06	−0.01	−0.03^***^	0.01	−3.79	−0.05	−0.02
MA						0.36^***^	0.02	15.67	0.31	0.40
SN	0.59^***^	0.02	24.44	0.54	0.63	0.19^***^	0.02	9.54	0.15	0.23
OB × SN	0.05^**^	0.01	6.00	0.04	0.07	0.02^***^	0.01	3.42	0.01	0.03
MA × SN						−0.07^***^	0.01	−8.12	−0.08	−0.05
Model	*R*	*R^2^*	*MSE*	*F*	*p*	*R*	*R^2^*	*MSE*	*F*	*p*
	0.70	0.49	0.46	287.62	0.000	0.85	0.73	0.17	480.67	0.000

*Bootstrap N = 5,000,*

*** *p < 0.001,*

** *p < 0.01,*

* *p < 0.05*.

Second, the moderating effect of perceptions of social norms was investigated. In model M_1_, the main effect of optimistic bias on message acceptance was significant (β = −0.04, *p* = 0.017, 95% CI = [−0.06, −0.01]); that is, the higher the optimistic bias, the lower the acceptance of health information. A significant interaction effect between optimistic bias and perceived social norms on message acceptance was detected (β = 0.05, *p* < 0.001, 95% CI = [0.04, 0.07]), indicating that perceived social norms have a negative moderating effect on the relationship between optimistic bias and message acceptance. The higher the perceptions of social norms, the smaller the negative effect of optimistic bias on message acceptance. In model M_2_, both optimistic bias (β = −0.03, *p* < 0.001, 95% CI = [−0.05, −0.02]) and message acceptance (β = 0.36, *p* < 0.001, 95% CI = [0.31, 0.40]) had a significant main effect on self-protection behavior; that is, optimistic bias has a notable negative impact and message acceptance has a notable positive impact on self-protection behavior. There was significant interaction between optimistic bias and perceived social norms (β = 0.02, *p* < 0.001, 95% CI = [0.01, 0.03]); that is, the higher the perceptions of social norms, the smaller the negative effect of optimistic bias on self-protection behavior. There was also significant interaction between message acceptance and perceived social norms (β = −0.07, *p* < 0.001, 95% CI = [−0.08, −0.05]); that is, the higher the perceptions of social norms, the smaller the effect of message acceptance on self-protection behavior.

Similar results are obtained by including gender, age, education, and occupation (occupied in healthcare or not) as control variables into the model: The regression coefficients of optimistic bias (β = −0.04, *p* < 0.001, 95% CI = [−0.05, −0.02]), message acceptance (β = 0.35, *p* < 0.001, 95% CI = [0.31, 0.40]), perceptions of social norms (β = 0.19, *p* < 0.001, 95% CI = [0.15, 0.22]), OB × SN (β = 0.02, *p* < 0.001, 95% CI = [0.01, 0.03]), and MA × SN (β = −0.06, *p* < 0.001, 95% CI = [−0.08, −0.05]) are all significant. The regression coefficients of all the control variables are not significant, except for age (β = 0.003, *p* = 0.025, 95% CI = [0.0003, 0.005]). This demonstrates the robustness of this conditional process model to a certain extent.

The three interactions in models M_1_ and M_2_ were all significant, indicating the significant moderating effect of perceived social norms between optimistic bias and message acceptance, between message acceptance and self-protection behavior, and between optimistic bias and self-protection behavior. These findings confirm H3 and verify the proposed conditional process model. Next, the direct and indirect effects of optimistic bias at different levels of perceived social norms (*M* ± 1SD) were analyzed. The results are shown in Table 4. When the value of social norms is one standard deviation below the mean, the confidence intervals of both direct and indirect effects do not include 0; that is, optimistic bias has significant direct and indirect effects on self-protection behavior. When the value of social norms is equal to the mean, the direct effect is significant and the indirect effect is not significant. When the value of social norms is one standard deviation higher than the mean, the confidence intervals include 0, which means the direct and indirect effects of optimistic bias on self-protection behavior are not significant. In other words, the effect of optimistic bias on self-protection behavior, as well as its indirect effect through message acceptance, becomes insignificant when individuals perceive strong social norms.

**Table 4:** Direct and indirect effects of optimistic bias moderated by social norms on self-protection behavior.

	**Social norms**	**Effect**	***SE***	**LLCI**	**ULCI**
	−1.01	−0.06	0.01	−0.08	−0.04
Direct effect	0.00	−0.03	0.01	−0.05	−0.02
	0.70	−0.02	0.01	−0.04	0.02
	−1.01	−0.04	0.01	−0.06	−0.01
Indirect effect	0.00	−0.01	0.01	−0.03	0.001
	0.70	0.01	0.01	−0.01	0.01

Furthermore, the moderating effect of perceived social norms was quantitively analyzed using the Johnson-Neyman technique. The results indicate that higher perceptions of social norms are associated with a smaller direct effect of optimistic bias on self-protection behavior. When perceptions of social norms are >6.94, the confidence interval of the direct effect of optimistic bias includes 0, and thus, the direct effect is not significant. In other words, optimistic bias has a significant negative impact on self-protection behavior when perceptions of social norms are <6.94. This impact decreases as the perceptions of social norms increase, and becomes insignificant when the perceptions of social norms are >6.94.

The indirect effect of optimistic bias on self-protection behavior can be divided into two paths: (1) optimistic bias—message acceptance; and (2) message acceptance—self-protection behavior. The moderating effect of perceived social norms on these two paths was examined, respectively. As for the first path, the negative impact of optimistic bias on message acceptance decreases with higher perceptions of social norms and becomes insignificant when the perceptions of social norms are >6.41. As for the second path, the impact of message acceptance on self-protection behavior increases with higher perceptions of social norms and remains significant in the whole range of social norm perceptions. These findings suggest that optimistic bias affects self-protection behavior both directly and indirectly through message acceptance, but both in a conditional way. The direct and indirect effects of optimistic bias on self-protection behavior are not significant when perceptions of social norms are very strong.

To sum up, the moderating effect of perceptions of social norms is mainly manifested in the following two ways: (1) The perceived social norms significantly decrease the negative effect of optimistic bias on self-protection behavior when within a certain range; that is, the higher the perceptions of social norms, the smaller the negative effect of optimistic bias. This effect is significant both in the direct path and the indirect path through message acceptance. (2) The negative effect of optimistic bias becomes insignificant at very high perceptions of social norms. However, message acceptance always affects self-protection behavior.

### Regional Differences

In addition, the differences in the moderating effect of perceived social norms between the three cities with different levels of risk were investigated. The results are shown in Table 5. In the low-risk area (Jinan, JN), social norms only play a significant moderating role between message acceptance and self-protection behavior. In the medium-risk area (Hangzhou, HZ), social norms play a significant moderating role between optimistic bias and message acceptance, and between optimistic bias and self-protection behavior. In the high-risk area (Wuhan, WH), social norms play a significant moderating role in all the three paths. It implies that the moderating role of social norms may become stronger as the level of risk increases. Thus, H4 is also confirmed. For further analysis, the three cities were coded according to the level of risk (JN = 1, HZ = 2, WH = 3). The second-order moderating effects of different cities on social norms were analyzed using PROCESS 3.5 (Preacher and Hayes, 2004)^4^. As shown in Table 6, the OB × SN × C coefficient in M_3_ is significant. It indicates that there are significant differences in the moderating effect of perceived social norms on optimistic bias—message acceptance as the level of risk increases. In M_4_, the OB × SN × C coefficient is significant and positive, and the OB × SN coefficient is positive. It indicates that the higher the risk, the greater the interaction between social norms and optimistic bias, that is, the greater the effect of social norms in decreasing the negative impact of optimistic bias. The MA × SN × C coefficient is significant and positive, and the MA × SN coefficient is negative. It indicates that the higher the risk, the smaller the interaction between social norms and message acceptance, that is, the smaller the effect of social norms in decreasing the impact of message acceptance on self-protection behavior.

**Table 5:** The moderating role of social norms in cities with different risk levels.

**Moderation path**	**Jinan (*****N*** **=** **308)**			**Hangzhou (*****N*** **=** **302)**			**Wuhan (*****N*** **=** **286)**		
	**Coefficients**	***SE***	**SN interval**	**Coefficients**	**SE**	**SN interval**	**Coefficients**	***SE***	**SN interval**
OB → MA	0.01	0.01	NULL	0.10^***^	0.01	<6.14	0.08^***^	0.02	<5.69
OB → SB	0.001	0.001	NULL	0.09^***^	0.02	<6.61	0.05^**^	0.01	<4.90
MA → SB	−0.09^***^	0.01	ALL	0.006	0.02	NULL	−0.04^*^	0.02	ALL

*** *p < 0.001,*

** *p < 0.01,*

* *p < 0.05. Coefficient is the regression coefficient of the interaction between the independent variable of the corresponding path and SN. SN interval is the value range of SN when the corresponding path coefficient is significant at the 0.05 level*.

**Table 6:** Second-order moderating effects.

**Variables**	**M**_****3****_ **(dependent variable: message acceptance)**					**M**_****4****_ **(dependent variable: self-protection behavior)**				
	**Coefficients**	***SE***	***t***	**LLCI**	**ULCI**	**Coefficients**	***SE***	***t***	**LLCI**	**ULCI**
Constant	−0.03	0.02	−1.39	−0.08	0.01	0.04^*^	0.02	2.18	0.04	0.07
OB	−0.02	0.01	−1.26	−0.05	0.01	−0.02^*^	0.01	−2.49	−0.04	−0.01
MA						0.33^***^	0.02	13.39	0.28	0.37
SN	0.57^***^	0.03	22.09	0.52	0.62	0.17^***^	0.02	7.61	0.12	0.21
OB × SN	0.06^***^	0.01	6.77	0.04	0.08	0.04^***^	0.01	5.23	0.02	0.05
MA × SN						−0.06^***^	0.02	−6.98	−0.07	−0.04
City (C)	−0.07^*^	0.03	−2.51	−0.13	−0.02	−0.11^***^	0.02	−5.31	−0.15	−0.07
OB × C	−0.01	0.02	−0.41	−0.04	0.03	0.01	0.01	1.36	−0.01	0.04
SN × C	−0.17^***^	0.03	−5.58	−0.23	−0.11	−0.05	0.03	−1.75	−0.03	0.08
MA × C						0.03	0.03	0.89	−0.10	0.01
OB × SN × C	0.03^*^	0.01	2.37	0.01	0.05	0.02^**^	0.01	2.77	0.01	0.04
MA × SN × C						0.03^**^	0.01	2.96	0.01	0.05
Model	*R*	*R^2^*	*MSE*	*F*	*p*	*R*	*R^2^*	*MSE*	*F*	*p*
	0.72	0.51	0.44	133.48	0.000	0.86	0.74	0.17	234.20	0.000

*Bootstrap N = 5,000,*

*** *p < 0.001,*

** *p < 0.01,*

* *p < 0.05*.

Furthermore, the direct and indirect effects of optimistic bias on self-protection behavior in the three cities under different levels of social norms were analyzed. As shown in Table 7, the direct effect of optimistic bias on self-protection behavior is significant in all the three cities when social norms are low (*M* – 1*SD*), insignificant in all the three cities when social norms are high (*M* + 1*SD*), and significant in Jinan and Hangzhou and insignificant in Wuhan when social norms are equal to the mean. This means that moderate social norms are sufficient to effectively reduce the negative impact of optimistic bias on self-protection behavior in the high-risk city, Wuhan. As shown in Table 8, it is only in Hangzhou and Wuhan that the indirect effect of optimistic bias is significant when social norms are low. The indirect effect of optimistic bias on self-protection behavior by reducing message acceptance is not significant in the low-risk area (Jinan).

**Table 7:** The impact of social norms on the direct effect of optimistic bias in cities with different risk levels.

**Social norms**	**Jinan**				**Hangzhou**				**Wuhan**			
	**Effect**	***SE***	**LLCI**	**ULCI**	**Effect**	***SE***	**LLCI**	**ULCI**	**Effect**	***SE***	**LLCI**	**ULCI**
−1.01	−0.05^***^	0.01	−0.08	−0.02	−0.06^***^	0.01	−0.08	−0.04	−0.07^***^	0.01	−0.09	−0.04
0.00	−0.03^**^	0.01	−0.06	−0.01	−0.02^*^	0.01	−0.04	−0.01	−0.01	0.01	−0.04	0.01
0.70	−0.02	0.01	−0.05	0.01	0.003	0.01	−0.02	0.02	0.03	0.02	−0.01	0.06

*** *p < 0.001,*

** *p < 0.01,*

* *p < 0.05*.

**Table 8:** The impact of social norms on the indirect effect of optimistic bias in cities with different risk levels.

**Social norms**	**Jinan**				**Hangzhou**				**Wuhan**			
	**Effect**	***SE***	**LLCI**	**ULCI**	**Effect**	***SE***	**LLCI**	**ULCI**	**Effect**	***SE***	**LLCI**	**ULCI**
−1.01	−0.02	0.02	−0.06	0.03	−0.03	0.01	−0.05	−0.01	−0.04	0.02	−0.07	−0.01
0.00	−0.01	0.01	−0.02	0.01	−0.01	0.01	−0.02	0.01	−0.01	0.01	−0.04	0.02
0.70	0.004	0.01	−0.01	0.02	0.01	0.01	−0.01	0.02	0.01	0.02	−0.03	0.04

## Discussion

### Implications

The frequent occurrence of public health crises, such as H1N1 influenza and COVID-19, in the twenty first century underscores a need to better understand individual health decisions in order to provide a theoretical basis for effective policy intervention. Among the many factors affecting health behavior, optimistic bias has received widespread attention. Unlike previous studies that solely investigate the relationship between optimistic bias and health behavior from the individual perspective (e.g., Williams and Clarke, 1997; Arnett, 2000; Bränström et al., 2006; Caponecchia, 2010; Popova and Halpern-Felsher, 2016; Masiero et al., 2018; Lopez and Leffingwell, 2020), this study introduces social norms from the group perspective to help better understand this relationship from the following aspects.

This study finds that people have internal and external reference points when deciding whether to adopt self-protection behaviors. Optimistic bias is the internal reference point in individuals' decisions to adopt health behaviors. Some authors believe that optimistic bias is a stable trait, and that it is through optimistic bias that individuals evaluate health information and decide whether to adopt health behaviors (Weinstein and Klein, 1995; Myers and Brewin, 1996; Helweg-Larsen, 1999; Radcliffe and Klein, 2002; Cho et al., 2013). This view implies that whether an individual adopt self-protection behaviors is a relatively independent decision. However, as demonstrated in this study, participants were obviously affected by social norm information (that is, how people around or close to me act) when deciding whether to adopt self-protection behaviors. This means that in addition to the individual-level reference point, there was also an external reference point, that is, perceived social norms, for participants when making health behavior decisions. Specifically, the negative effect, both directly and indirectly through message acceptance, of optimistic bias on self-protection behavior decreased as the perceptions of social norms increased within a certain range. This negative effect disappeared when the perceptions of social norms approached the highest value. In indicates that no matter how high the optimistic bias is, it would not become an obvious obstacle to health behavior, as long as the perceptions of social norms are strong enough. In other words, in this case, the external reference point may completely replace the internal reference point and become the key to individual health decisions. It should be noted that social norms play a different moderating role in the direct and indirect paths through which optimistic bias affects health behavior. When the perceptions of social norms are extremely high, both the direct and indirect effects of optimistic bias on health behavior are not significant. When the perceptions are at an average level, the direct effect is still significant, while the indirect effect is insignificant. This may be because optimistic bias mainly affects health behavior through the direct path when the perceptions of social norms are not high enough.

The discovery of internal and external reference points in individual health decision-making has both theoretical and practical significance. From a theoretical point of view, this finding addresses a contradiction in previous studies to a certain extent: most studies in this area believe that optimistic bias has a significant negative effect on health behaviors (Weinstein and Klein, 1995; Harris and Napper, 2005; Park and Ju, 2016; Hwang et al., 2019). However, a few other studies have reported that optimistic bias did not affect the health behaviors of participants (Taylor and Gollwitzer, 1995; Cho et al., 2013). One possible explanation for this contradiction is that one or some of the different health behaviors examined in previous studies contain strong normative information in themselves, which significantly reduced the negative effect of optimistic bias on health behavior (which might not be realized by the experimenter or the participants). However, normative perception has been ignored in previous studies to a certain extent. In other words, the findings of this study provide a new perspective: do different behaviors themselves convey different levels of normative information? Addressing this new question should offer important insights for a better understanding of the relationship between optimistic bias and health behavior. On the other hand, this discovery has important practical significance for formulating effective policy interventions. Previous studies have shown that self-affirmation effectively increased the self-efficacy of participants, thereby reducing optimistic bias and improving health behaviors (Klein et al., 2010; Epton et al., 2015; Lü and Zhao, 2017). The existence of dual reference points means that the negative impact of optimistic bias on health behavior can also be mitigated by intervening in individual perceptions of social norms. Compared with self-affirmation, it is easier and more cost-effective to manipulate individual perceptions of social norms.

The analysis of data from three different cities further refines our understanding of the moderating role of social norms in the relationship between optimistic bias and health behavior. The analysis results show that the higher the risk, the more significant the role of social norms, which is mainly manifested in two ways: First, as the risk of infection increases, the paths in which social norms work increase notably. In the low-risk area (Jinan), social norms mainly affect self-protection behavior through message acceptance. In the high-risk area (Wuhan), the role of social norms is not only reflected in the indirect path (OB → MA → SB), but also in the direct path (OB → SB). Second, the increase in the risk of infection expands the boundary conditions for social norms to suppress the negative effect of optimistic bias. In other words, strong normative information is needed to suppress the negative effect of optimistic bias in the low-risk area. However, moderate normative information is sufficient to achieve similar results in the high-risk area. This finding has important practical significance for the world severely affected by COVID-19 pandemic, because it means that social norms can play the most effective role where they are most needed. Normative information can be delivered to the public indiscriminately through social norms campaigns, or to some individuals in the form of personal normative feedback (Blanton et al., 2008). These two ways can also be combined to achieve the best results.

### Limitations

As with any research, this study has some limitations. First, this is a correlation study based on cross-sectional data. Therefore, definite conclusions cannot be drawn on the causal relationship between related variables based on the findings of this study. In this sense, although SNA offers important theoretical insights to this study, it does not meet the SNA standards (Dempsey et al., 2018): the effects of normative information intervention were not evaluated by experimental investigation. Second, as pointed out in the introduction, social norms can generally be divided into descriptive and injunctive norms. However, this research only focuses on the role of descriptive norms in moderating the relationship between optimistic bias and self-protection behavior. Future studies can continue to refine this research by including injunctive norms. Third, in addition to optimistic bias and perceptions of social norms that were assessed in this study, there are many factors that affect self-protection behavior, such as subjective risk perception, perception of susceptibility to COVID-19 infection, and motivation to adopt risk-reduction behaviors. Taking them into consideration will further improve the reliability of our conclusions. Forth, Wuhan, Hangzhou, and Jinan were classified into high-, medium-, and low-risk areas based on the magnitude of the number of confirmed cases. However, the gap between Wuhan and Hangzhou was much larger than that between Hangzhou and Jinan in terms of absolute numbers, which may cause the difference between Hangzhou and Jinan to be insignificant. Fifth, knowledge was used as a marker variable to assess common method bias. However, what was measured is the perception of knowledge in terms of item content. Future studies should distinguish between these two concepts to improve research quality. Finally, the online data collection excluded individuals who did not have Internet access (e.g., some elderly people) from the sample. However, the elderly are most susceptible to COVID-19. This may create a bias in the data.

## Data Availability Statement

The raw data supporting the conclusions of this article will be made available by the authors, without undue reservation.

## Ethics Statement

The studies involving human participants were reviewed and approved by IRB of School of Economics and Management, Zhejiang University of Science and Technology. The patients/participants provided their written informed consent to participate in this study.

## Author Contributions

SC conceived this study, designed questionnaires, collected data, and wrote introduction. HH analyzed data and wrote method and result sections. JL wrote discussion and finalized the manuscript for submission. All authors contributed to the article and approved the submitted version.

## Conflict of Interest

The authors declare that the research was conducted in the absence of any commercial or financial relationships that could be construed as a potential conflict of interest.

## Footnotes

^1^See https://edition.cnn.com/2020/03/24/health/kentucky-coronavirus-party-infection/index.html.

^2^See https://www.who.int/emergencies/diseases/novel-coronavirus-2019/events-as-they-happen.

^3^In the custom model, bmatrix = 1, 1, 1; wmatrix = 1, 1, 1.

^4^In the custom model, bmatrix = 1, 1, 1; wmatrix = 1, 1, 1; zmatrix = 1, 1, 1; wzmatrix = 1, 1, 1.
